# Common mouse models of tauopathy reflect early but not late human disease

**DOI:** 10.1186/s13024-023-00601-y

**Published:** 2023-02-02

**Authors:** Kathrin Wenger, Arthur Viode, Christoph N. Schlaffner, Patrick van Zalm, Long Cheng, Tammy Dellovade, Xavier Langlois, Anthony Bannon, Rui Chang, Theresa R. Connors, Derek Oakley, Bernhard Renard, Juri Rappsilber, Bradley Hyman, Hanno Steen, Judith A. Steen

**Affiliations:** 1 F.M. Kirby Neurobiology Center, Department of Neurobiology, Boston Children’s Hospital and Harvard, Medical School; Center for Life Science, RM 12030, 3 Blackfan Circle, Boston, MA 02115 USA; 2 Departments of Pathology, Boston Children’s Hospital and Harvard Medical School, Boston, MA USA; 3 Data Analytics and Computational Statistics, Hasso-Plattner-Institute, Faculty of Digital Engineering; University of Potsdam, Potsdam, Germany; 4 AbbVie, Cambridge Research Center, Cambridge, MA USA; 5 Massachusetts Alzheimer’s Disease Research Center, Massachusetts General Hospital, Harvard Medical School, Boston, MA USA; 6 Bioanalytics, Institute of Biotechnology, Technische Universität Berlin, Berlin, Germany; 7 Neurobiology Program, Boston Children’s Hospital, Boston, MA USA

**Keywords:** Alzheimer’s disease, Human Tau, Post-translational modifications, Protein aggregation, Tauopathy, Mouse model, Disease progression, Quantitative proteomics, P301S, P301L

## Abstract

**Background:**

Mouse models that overexpress human mutant Tau (P301S and P301L) are commonly used in preclinical studies of Alzheimer’s Disease (AD) and while several drugs showed therapeutic effects in these mice, they were ineffective in humans. This leads to the question to which extent the murine models reflect human Tau pathology on the molecular level.

**Methods:**

We isolated insoluble, aggregated Tau species from two common AD mouse models during different stages of disease and characterized the modification landscape of the aggregated Tau using targeted and untargeted mass spectrometry-based proteomics. The results were compared to human AD and to human patients that suffered from early onset dementia and that carry the P301L Tau mutation.

**Results:**

Both mouse models accumulate insoluble Tau species during disease. The Tau aggregation is driven by progressive phosphorylation within the proline rich domain and the C-terminus of the protein. This is reflective of early disease stages of human AD and of the pathology of dementia patients carrying the P301L Tau mutation. However, Tau ubiquitination and acetylation, which are important to late-stage human AD are not represented in the mouse models.

**Conclusion:**

AD mouse models that overexpress human Tau using risk mutations are a suitable tool for testing drug candidates that aim to intervene in the early formation of insoluble Tau species promoted by increased phosphorylation of Tau.

**Supplementary Information:**

The online version contains supplementary material available at 10.1186/s13024-023-00601-y.

## Background

Advancements in medicine have extended the human life span resulting in an aging population prone to dementia for which there is currently no cure available. Alzheimer’s Disease (AD) is the most common type of dementia and is responsible for 60–70% of all cases. One important hallmark of AD is the appearance of tangles made of aggregated Tau protein in later symptomatic stages [1, 2]. The abundance of the Tau tangles correlates with neuronal death and cognitive decline as the pathological Tau is neurotoxic [3–5]. Due to the tight correlation between Tau pathology and cognitive decline, the removal of pathological forms of Tau has become an important drug development strategy. From the analysis of human brain specimens of AD patients, it is known that pathological Tau is highly modified and that these modifications result in structural changes. The most common post-translational modification (PTM) of AD pathological Tau is phosphorylation. But ubiquitination, acetylation, and cleavage of the Tau protein have also been identified as important to pathology and structure [6–9]. Currently, no Tau-targeting drug candidate is FDA approved. All previous drug candidates failed in clinical studies due to a lack of efficacy in humans, despite showing efficacy in preclinical mouse studies. This leads us to the question – to which extent do these mouse models represent the human disease, in particular human Tau pathology, at the molecular level?

**Fig. 1:**
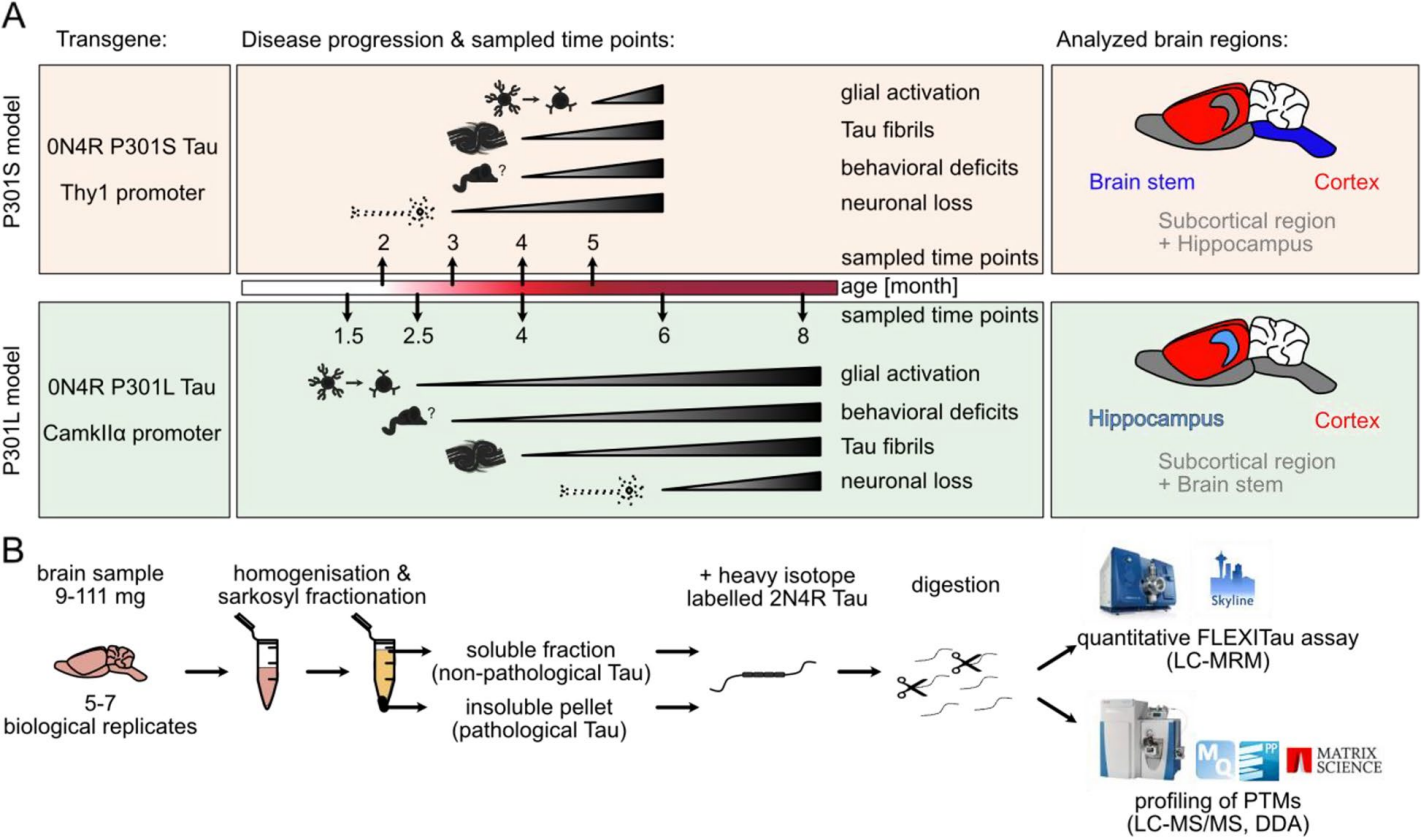
Overview on pathological and behavioral changes of the P301S and the P301L mouse model. **A** Overview on the disease progression of the P301S and the P301L mouse model and the respective analyzed brain regions and time points [11–18]. **B** Schematic overview of the sample preparation and PTM-focused proteomic workflow

To answer this question, we conducted an in-depth analysis of two commonly used AD mouse models, the Thy1-hTau.P301S (P301S) and the rTg(tauP301L)4510 (P301L) mouse model. These models induce the formation of Tau pathology through the neuronal expression of transgene human Tau with risk mutations mostly associated with familial cases of early-onset frontotemporal dementia (P301S and P301L) [10]. Besides Tau pathology, the mice also exhibit glial activation, neuronal loss, and behavioral deficits thereby mimicking phenotypes observed in human AD (Fig. 1A) [11–18]. While antibody-based immunohistochemistry approaches targeting a couple of Tau modifications have contributed significantly to understanding the Tau distribution and the appearance of tangles [6, 19, 20], it is not known if the molecular mechanisms of Tau aggregation are the same as in humans, where we observe an ordered accumulation of PTMs as AD progresses. A comprehensive mapping of Tau PTMs in the P301S and the P301L mouse models would provide information regarding advantages and shortcomings of the mouse models and how to use them more effectively. Also, a detailed comparison to human dementia patients carrying the P301L Tau mutation and human AD patients is elusive. To obtain a temporally and spatially resolved overview of the molecular features of Tau, brain samples over the range of disease progression of affected and unaffected brain regions were analyzed for both mouse models. The results were compared to published data from human AD [20]. Additionally, samples from a human cohort of patients carrying the P301L mutation were analyzed for comparison. Insoluble (Tau aggregates) and soluble Tau fractions were obtained from brain tissue using detergent-based (sarkosyl) fractionation (Fig. 1C). A qualitative and quantitative mass spectrometry (MS)-based proteomics approach was used to identify PTMs of Tau as well as their modification extent.

## Materials and methods

### Animals

P301S mice (Thy1-hTau.P301S (CBA.C57BL/6)) that were used for this study were homozygous for the MAPT*P301S transgene. The original breeder P301S mice were obtained from Medical Research Council (MRC) under licensing agreement. P301L mice [Tg(CamK2a-tTA)1Mmay Fgf14/Tg (tet0 MAPT*P301L) 4510Kha] were bigenic carrying a single copy of the CaMKIIα-tTA transgene and the MAPT*P301L transgene.

The P301L mice were generated by crossing animals hemizygous for the CaMKIIα-tTA transgene purchased from Jackson Laboratories (Stock Number 007004) to animals hemizygous for the MAPT*P301L transgene purchased from Jackson Laboratories under licensing agreement with Mayo Clinic (Stock Number 015815). Breeders for both lines were sent to Charles River Laboratories, Wilmington, MA for establishing and maintenance of mouse colonies as well as genotyping. Adult mice were then delivered to the AbbVie Cambridge Research Center (CRC) for use in studies. Once in the CRC vivarium, P301S and P301L mice were group-housed in individually ventilated cages (Innovive, San Diego, CA, USA) on a 12 h:12 h light–dark cycle and provided ad libitum access to food and water. All procedures conducted on animals at the CRC were approved by the AbbVie Institutional Animal Care and Use Committee (IACUC). To eliminate sex differences, only female mice were used for the study.

### Tissue collection

To cover all stages of disease 5–7 biological replicates of 4–5 time points distributed over the whole disease progression were analyzed. This includes 2, 3, 4, and 5 months of age for the P301S and 1.5, 2.5, 4, 6, and 8 months of age for the P301L model. Mice were euthanized with sodium pentobarbital and perfused with 0.1 M phosphate-buffered saline. The brains were rapidly removed and right hemisphere per animal was dissected into selected brain regions. The brain regions collected, cortex and brainstem in P301S and cortex and hippocampus in P301L are known to show age-dependent increases in Tau pathology. For both models, the remaining unaffected regions, except for the cerebellum, were pooled and are referred to as the subcortical region. Tissues were placed in microfuge tubes and quickly frozen in liquid nitrogen. The left hemisphere was drop-fixed in 10% formalin for 24 h before being switched to 70% ethanol. Brains were stored in 70% ethanol until all brains from the study were collected to allow all samples to be processed for paraffin embedding at the same time (Leica).

### Human tissue samples

Frozen human post-mortem frontal gyrus (BA46) specimens from patients carrying the P301L Tau mutation and healthy non-demented age-matched control subjects were obtained from the Massachusetts Alzheimer’s Disease Research Center Brain Bank. The demographic characteristics of the subjects are shown in Table S1.

### Generation of heavy labeled 2N4R Tau standard

To quantify Tau amounts the FLEXITau workflow as previously described [21] was used. The heavy labeled Tau standard was transcribed and translated in vitro in a cell-free wheat germ expression system according to the manufacturer’s protocols (Cell Free Sciences, Wheat Germ Expression H Kit-NA) in the presence of heavy isotope ^13^C_6_-labeled lysine (+ 6) and ^13^C_6_,^15^N_4_-labeled arginine (+ 10). Afterward, the expressed Tau standard was dephosphorylated using Lambda Protein Phosphatase (New England Biolabs) according to the manufacturer’s instructions. Subsequent purification was performed using Ni-Sepharose beads (Ni-Sepharose High-Performance resin, GE Healthcare).

### Fractionation of human and murine brain tissue samples

Frozen tissue samples (9–111 mg) were homogenized in 5 volumes TBS buffer (50 mM Tris–HCl buffer, pH 7.4, containing 150 mM NaCl, 0.5 mM MgSO4, phosphatase inhibitor cocktail (Roche), protease inhibitor cocktail (Roche) and Trichlostatin A (2µM)), using a Precellys tissue homogenizer (5500 rpm). To separate the insoluble Tau species from soluble Tau sarkosyl fractionation was performed on the homogenate. Therefore, cell debris was removed by centrifugation at 14,000 rpm for 20 min at 4 °C. The supernatant was diluted 1:1 with 2 × salt/sucrose solution (1.6 M NaCl, 20% Sucrose, 20 mM Tris–HCl buffer, pH 7.4, 2 mM EGTA, phosphatase inhibitor cocktail (Roche), protease inhibitor cocktail (Roche) and Trichlostatin A (2µM)). This supernatant is referred to as soluble fraction 1. Soluble fraction 2 was derived by reextraction of the remaining pellet with 1 × salt/sucrose solution (0.8 M NaCl, 10% Sucrose, 10 mM Tris–HCl buffer, pH 7.4, 1 mM EGTA phosphatase inhibitor cocktail (Roche), protease inhibitor cocktail (Roche) and Trichlostatin A (2µM)) and centrifugation at 14,000 rpm for 20 min at 4 °C. Both soluble fractions were treated with sarkosyl (1% final concentration) for 1.5 h at room temperature. Afterwards soluble fractions 1 and 2 were pooled and ultracentrifuged at 50,000 rpm for 1.5 h at 4 °C. The supernatant was transferred to a new tube (sarkosyl-soluble fraction). The sarkosyl-insoluble pellet, which contains the aggregated Tau species, was carefully washed twice with 20 µL PBS and resuspended in PBS using sonification (QSonica, 20% amplitude, 3 × 20 s). The protein concentration in the extracts was determined by bicinchoninic acid assay (BCA Protein Assay Kit, Thermo Scientific). After adding equal amounts of dephosphorylated and purified heavy 2N4R Tau standard the insoluble fractions were diluted with 8 M urea and processed separately using Filter Aided Sample Preparation (FASP Protein Digestion Kit, Expedeon) with DTT as reduction agent and 1% acrylamide for cysteine alkylation. Protein mixtures were digested with trypsin overnight at 37 °C (sequencing grade modified trypsin, Promega, Madison, WI). Acidified peptides were desalted using C18 extraction tips (Nest). Vacuum-dried peptides were reconstituted in sample buffer (0.1% formic acid, 5% acetonitrile).

### FLEXITau measurement and analysis of human and murine sarkosyl fractions

Before Liquid chromatography-Selective Reaction Monitoring (LC-SRM) analysis, part of the resuspended sample was spiked with additional peptides. For the mouse models these included the light FLEX-peptide SENLYFQGDISR (15 fmol/µl final concentration), the heavy 0N Tau specific peptide STPTAEAEEAGIGDTPSL[+ 7]EDEAA[+ 4]GHVTQA[+ 4]R (50 fmol/µl final concentration) as well as the peptides carrying respective mutation (P301L: HVLGGGSVQIVYKPVDLSK[+ 8] or P301S: HVSGGGSVQIVYKPVDLSK[+ 8]). Human samples were spiked with heavy 0N, 1N (STPTAEAEEAGIGDTPSL[+ 7]EDEAA[+ 4]GHVTQA[+ 4]R), 3R (VQIVYKPVDLSK[+ 8]) and P301L Tau specific peptides (all spiked samples had a 50 fmol/µl final concentration) (all peptides were in QuantPro quality synthesized by Thermo Fischer). LC-SRM measurements of Tau L/H peptide ratios were performed as described previously [21]. After optimization of transitions using in-house DDA spectral libraries and heavy-isotope labeled Tau standards the samples were analyzed on a quadrupole mass spectrometer (5500 QTRAP, Sciex) which was coupled to an Eksigent micro-autosampler AS2 and a microflow pump (Eksigent/Sciex, Framingham, USA) as described above but operated at 5 µL/min. Here, 1.25 µg of peptides seperated on a 25 cm column (Proteocol C18G 200A˚, 250 mm × 300 μm ID Trajan Scientific and Medical, Australia) using a 25 min gradient from 0 to 35% acetonitrile. Three to five transitions were monitored for each precursor by SRM with a retention time window of 45 s and a target scan time of 0.5 s to ensure an optimal amount of data points per peak. SRM data were analyzed and validated in Skyline-daily (version 21.0.9.105, MacCoss Lab Software, University of Washington, Seattle, WA) [22]. In total 18 Tau peptides were quantified, and the L/H ratios of the peak area were exported for every peptide. The absolute abundance of Tau was calculated using the FLEX peptide L/H ratio and the L/H ratio of the peptide with the highest ratio that is shared between the mouse and the transgene human Tau as described before [23]. The modifications extent was calculated through normalization of the peptide L/H ratio to the shared or human-specific peptide with the highest L/H ratio, depending on the specificity of the peptide. Plotting, student’s t-test and Pearson correlation were done using GraphPad Prism 8 version 8.2.1 (GraphPad Software Inc.). Hierarchical cluster analysis using euclidean distance and complete linkage was performed in R (4.1.2) using R studio (2021.09.1) and the pheatmap (1.0.12) package [24, 25].

### LC–MS/MS of murine sarkosyl-insoluble fractions and data analysis

The remaining part of the sample buffer resuspended murine sample was used for LC–MS/MS analysis. Here a QExactive mass spectrometer (Thermo Fisher Scientific, Bremen) coupled to a micro-autosampler AS2 and a nanoflow HPLC pump (Eksigent, Dublin, CA) was used. Peptides were loaded on a capflow PicoChip column (150 mm × 10 cm Acquity BEH C18 1.7 mm 130 Å, New Objective, Woburn, MA) with 2 ml/min solvent A (water + 0.1% formic acid). The elution was performed by a 135 min gradient at a flow rate of 1 µl/min. Solvent B (acetonitrile + 0.1% formic acid) was increased from 2 to 20% over the first 110 min. Between 110 and 120 min, it was further increased to 30%. For the wash step solvent B was ramped up to 95% within 1 min and was kept constant at this percentage for 5 min. Afterwards, a re-eqilibration step at 2% B for 5 min was performed. During the whole run, the PicoChip containing an emitter for nanospray ionization was kept at 50 °C. A full mass spectrum with a resolution of 70,000 was acquired in a mass range of 375–1400 m/z (AGC target 3 × 10^6^, maximum injection time 60 ms). The 12 most intense precursor ions were selected for fragmentation via higher-energy c-trap dissociation (HCD, resolution 17,500, AGC target 5×10^4^, maximum injection time 100 ms, isolation window 1.6 m/z, normalized collision energy 27%). Once a precursor ion was picked for fragmentation it was excluded for the following 25 s.

To identify PTMs of Tau, the MS raw data were processed with ProteinPilot™ Software 5.02 (Paragon Algorithm 5.0.2.0.5174, Sciex), MaxQuant software version 1.6.5.0 [26], and Mascot using the Mascot Deamon version 2.6.0 (Matrix Science). QExactive raw files were converted into mgf data. Collected spectra were searched against a *mus musculus* proteome database including isoforms (21,215 entries, downloaded from uniprot.org on 06/10/2019) which was used in all three search engines. To avoid mismatching of PTMs all murine Tau isoforms were removed and the human 0N4R Tau (Uniprot ID: P10636-6) with the P301S (dbSNP ID: rs63751438) or P301L (dbSNP ID: rs63751273) mutation was added.

In ProteinPilot™ the following settings were applied: sample type ‘Identification’; Cys Alkylation ‘Propionamide’; Digestion ‘Trypsin’; instrument type ‘Orbi MS, Orbi MS/MS’; ‘thorough ID’ search mode; ‘ID focus on biological modifications’. A cutoff of 95% confidence was employed for all modified peptides. In addition, only phosphorylation of S, T and Y, methylation of K and R, acetylation of K and ubiquitination of K were considered.

Within the MaxQuant software, the following settings were used: trypsin (specificity set as Trypsin/P) with up to two missed cleavages and a minimum peptide length of 5 amino acids. Oxidation of M, acetylation of N-termini and K, phosphorylation of S and T, methylation of K and R and ubiquitination (GlyGly) of K were chosen as variable modifications and propionamide was set as static modification of cysteine with a maximum of three modifications per peptide. False discovery rate (FDR) was set to 1% on peptide and protein levels and was determined by searching a reverse database. Peptide identification by match between runs was disabled. For all other search parameters, the default settings were used.

The Mascot search was performed considering peptide charge states of 2 + , 3 + and 4 + including a 10 ppm tolerance. The MS/MS search was run with a mass tolerance of 0.6 Da. The search was performed with trypsin as the used enzyme allowing a maximum of 2 missed cleavages and Oxidation of M, acetylation of K, methylation of K and R, citrullination of R, phosphorylation of S, T and Y and ubiquitination (GlyGly) of K were chosen as variable modifications and propionamide was set as static modification of cysteine. Afterwards, the PTM results of different search algorithms were cumulatively combined. Hierarchical cluster analysis using euclidean distance and complete linkage was performed in R (4.1.2) using R studio (2021.09.1) and the pheatmap (1.0.12) package [24, 25].

### LC–MS/MS of human sarkosyl-insoluble fractions and data analysis

The remaining part of the sample buffer resuspended human sample was used for LC–MS/MS analysis. Here a timsTOF Pro mass spectrometer (Bruker Daltonics, Billerica, MA) coupled to a nano elute liquid chromatography (Bruker) was used. Peptides were loaded on a C18 UHPLC column 25 cm × 75 μm (1.6 µm particle size) from IonOpticks (Fitzroy, Australia). The elution was performed by a 120 min gradient at a flow rate of 0.4 µl/min. Solvent B (acetonitrile + 0.1% formic acid) was increased from 0 to 23% over the first 90 min. Between 90 and 100 min, it was further increased to 35%. For the wash step solvent B was ramped up to 80% within 10 min and was kept constant at this percentage for 10 min. During the whole run, the column was kept at 50 °C. For the data-dependent analysis, the mass spectrometer was operated in DDA-PASEF mode. 10 PASEF MS/MS scans were triggered per cycle. DDA-PASEF parameters were set as follow: m/z range 100–1700, mobility (1/K0) range was set to 0.60–1.6 V.s/cm2, the accumulation and ramp time were of 100 ms. Target intensity per individual PASEF precursor was set to 20,000. The values for mobility-dependent collision energy ramping were set to 59 eV at an inversed reduced mobility (1/K0) of 1.6 V.s/cm2 and 20 eV at 0.6 V.s/cm2. Collision energies were linearly interpolated between these two 1/K0 values. The acquired data was converted to mgf using the Compass data analysis software (Bruker, version 5.3).

For identification spectra were searched against a canonical *homo sapiens* proteome database (20,370 entries, downloaded from uniprot.org on 11/24/2020) which was used in all search engines. To avoid mismatching of PTMs all 6 human Tau isoforms and all 4R Tau isoforms including the P301L (dbSNP ID: rs63751273) mutation were added to the database.

Identification of peptides was performed using the Mascot and Fragpipe search algorithm. The Mascot search was performed the same way as the murine samples. The Fragpipe search included the MSFragger, Philosopher and IonQuant modules [27–30]. MSFragger 3.4 was ran using the standard settings. Oxidation of M, acetylation of K, methylation of K and R, phosphorylation of S, T and Y and ubiquitination (GlyGly) of K were chosen as variable modifications and propionamide was set as static modification of cysteine. Philosopher 4.1.1 was used for statistical validation of identified peptides. IonQuant 1.7.17 was used for quantification where a minimum of 1 ion was used for peptide quantification. Afterwards, the PTM results from different search algorithms were cumulatively combined. Hierarchical cluster analysis using euclidean distance and complete linkage was performed in R (4.1.2) using R studio (2021.09.1) and the pheatmap (1.0.12) package [24, 25].

### Tissue processing and AT100 immunoreactivity

Three to four brains from different treatment groups were embedded together into paraffin blocks (Sakura Finetek, USA) in sagittal plane. Five µm paraffin sections were collected through the entire brain and mounted onto a glass slides. Matched sections through the cortex and brainstem for P301S mice and cortex and hippocampus for the P301L mice were stained for AT100 immunoreactivity using the BOND RX stainer with the Refine Detection kit (Leica). In brief, the slides were deparaffinized, rehydrated, and placed on the BOND RX where they underwent a series of pretreatments including peroxide block (Leica) 5% donkey serum blocking (017–000-121, Jackson ImmunoResearch Labs), mouse IgG blocking (MKB-2213, Vector laboratories) and were then incubated overnight at 4 C° in the mouse monoclonal antibody to AT100 tau (MN1060, Thermo Fisher) at a concentration of 0.006 µg/mL. Slides were then returned to the BOND RX where they underwent series of washes before being incubated in Biotin-SP-conjugated F(ab')2 donkey anti mouse IgG(H + L) secondary antibody (715–006-151, Jackson ImmunoResearch Labs, West Grove, PA, USA) at a concentration of 2 µg/mL for 20 min. After additional washing steps, the slides were incubated for 15 min in Streptavidin/Horseradish Peroxidase (RE7104, Leica) and the immunoreactivity was visualized using diaminobenzidine (DAB; Leica, Buffalo Grove, IL, USA) and counterstained with hemotoxylin (Leica).

### AT100 immunoreactivity quantification

For both studies, 3 matched sections per brain were analyzed using the Area Quantification module in HALOTM image analysis software (Indica Labs, Albuquerque, NM, USA). For P301s and P301L mice, the entire cortex was outlined as the region of interest. Using the software, the "threshold" was determined by an observer who was blind to the age of the animals. The threshold was set so that the positive brown DAB stain of the AT100 immunoreactivity was recognized by the software and the background/non-specific staining was excluded from the analysis. Once an appropriate threshold was set, the software measured the percentage of the area of interest (cortex) containing the positive immunoreactivity (AT100). The percentages for the 3 sections measured per brain were then averaged to obtain a single value for each animal.

## Results

### Insoluble Tau increases during progression with different dynamics between specific brain regions

**Fig. 2:**
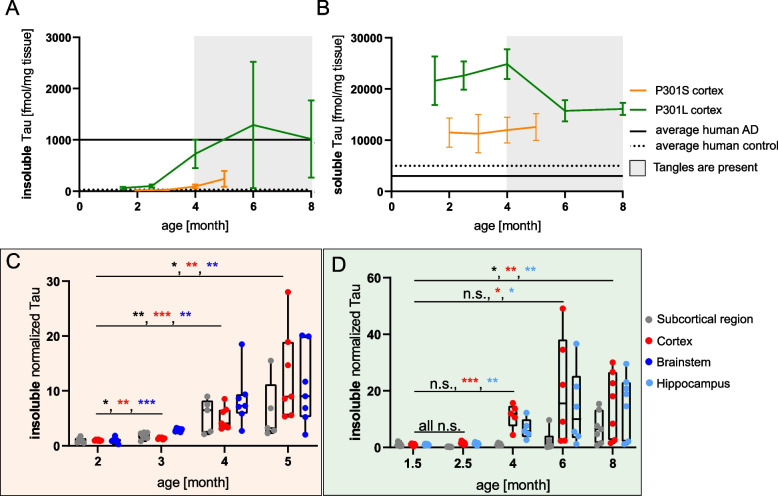
Quantification of insoluble and soluble Tau fractions in the P301S and the P301L model. **A**, **B** Average absolute amount of cortical insoluble (**A**) and soluble (**B**) Tau for both mouse models compared to average levels of human AD (BA39) (mean +—SD) (human data adapted from [20]). **C**, **D** Relative amount of insoluble total Tau measured in specified brain region compared to the first time point of the P301S (**C**) and the P301L (**D**) model (Significance was determined using a t-test, 5–7 replicates for each condition)

Measurements of sarkosyl insoluble Tau abundance reveal the progression of disease and dynamics of insoluble Tau accumulation in the mouse models. Cortical insoluble Tau is detectable at the first two time points, showing that insoluble Tau species form long before tangle formation is observable by immunostaining described in the literature at 4 months in both models (Fig. 2A) [13, 31].

The appearance of Tau tangles coincides with a 5.1 (P301S) and 11.2 (P301L) fold increase in the cortical amount of insoluble Tau at the 4 month time point (Fig. 2C and D). Beyond this initial increase, P301S displays a minimal increase, whereas P301L shows an abrupt increase with high variation between animals (Fig. 2A). To determine if the high variation observed for the P301L animal model is intrinsic to the model or caused by other factors such as the sample preparation and the detection method, we performed AT100 immunostaining on the remaining brain hemisphere. The orthogonal measurement method showed the same insoluble tau variability shown by mass spectrometry (Fig. S1A). In addition, the absolute amount of insoluble Tau as derived from the FLEXITau assay and AT100 immunoreactivity correlate tightly in both mouse models (Pearson correlation > 0.87) (Fig. S1B and S1C), suggesting that the variation is a property of the animal model.

At late stages the amount of insoluble Tau in the P301L model is comparable to symptomatic human AD patients (Braak stage IV-V) (~ 1000 fmol/mg), while the P301S model shows 4 × less insoluble Tau (~ 250 fmol/mg). The amount of insoluble Tau has been shown to correlate with neuronal loss and behavioral deficits [15, 19].

Cortical levels of soluble Tau are high in both models due to the overexpression of the transgene Tau, which is influenced by the promoter and the zygosity. In comparison to Tau in healthy human controls, the mouse models show overexpression of 2.4x (P301S, 12,000 fmol/mg) or 4.6x (P301L, 23,000 fmol/mg) (Fig. 2B).

Insoluble Tau shows different accumulation dynamics in the two mouse models across brain regions. In the P301S model, the brainstem shows the highest and fastest increase in insoluble Tau followed by the cortex and the subcortical region (Fig. 2C). If insoluble Tau accumulation is used as a measure of disease progression, the cortex and the hippocampus are the fastest progressing brain regions in the P301L model, whereas the subcortical region shows delayed pathology and only starts at 6 months of age (Fig. 2D). These measurements suggest a role for the promotors in the mouse models in driving Tau pathology (P301S animals -Thy1.2; P301L animals CaMKIIα). Thus, downstream effects of Tau pathology including synaptic loss, neuronal death and cognitive impairment mirror Tau promoter-driven expression patterns. Thus, the distribution of Tau pathology in the mouse brain is different from human AD, where Tau pathology spreads throughout the brain in an ordered progression, inducing pathology in connected brain regions [5, 32].

In the analyzed mouse models amyloid-β (Aβ) pathology is absent as no Aβ specific peptides were identified in the insoluble and soluble fraction. The amyloid precursor protein (APP) was exclusively identified in the soluble fraction and its amount remains constant in both mouse models throughout disease progression (Fig. S2A and S2B).

### Phosphorylation, citrullination, ubiquitination and methylation are observed modifications of insoluble Tau in the P301S and P301L mouse models

**Fig. 3:**
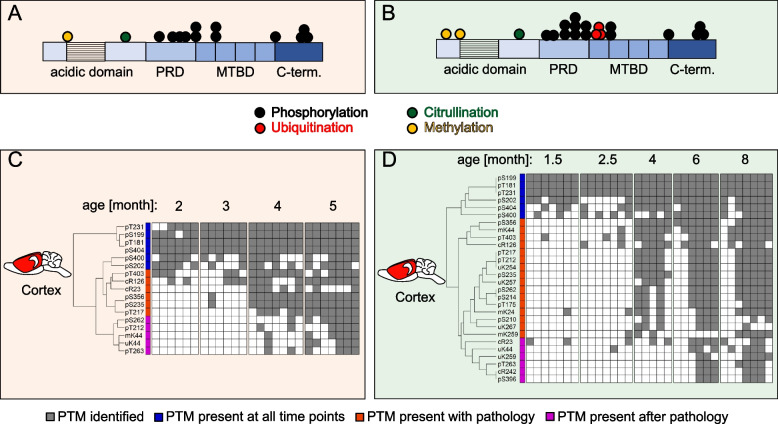
PTMs in insoluble Tau occur in a sequential manner during disease progression in mouse models. **A**, **B** A summary of PTMs identified in cortical insoluble Tau at the last time point of the P301S (**A**) and the P301L (**B**) models. PTMs represented are found in $\ge$ 50% of the biological replicates. **C**, **D** A sequential addition of PTMs in the cortex during disease progression is observed with unbiased Euclidean distance hierarchical clustering of Tau PTM data (binary—presence/absence) from the P301S (**C**) and the P301L (**D**) models. PTMs used for clustering were identified in ≥ 50% of the biological replicates. Grey squares indicate the presence of a PTM. Annotated clusters represent PTMs identified at all time points (blue), PTMs appearing concomitant with tangle formation (orange), and PTMs that appear after tangle formation (magenta)

In humans, AD pathological species of Tau are heavily post translationally modified and display phosphorylation, ubiquitination and acetylation. To understand which Tau PTMs contribute to Tau aggregation in the mouse models, Tau PTMs were mapped using an untargeted proteomics approach across all time points and brain regions. Phosphorylation, citrullination, methylation, and ubiquitination of Tau were identified and localized in both models. Figure 3A and 3B display the PTMs identified with a frequency above 50% in the cortex at the end stage of disease for each model. Our analysis shows that the main PTM driving the aggregation of transgene human Tau in the mouse models is phosphorylation (12–15 sites) concentrated within the Proline-rich domain (PRD) and the C-terminus. In addition, a few methylation and citrullination sites (1–3 sites) were found within the acidic domain. The P301L model exhibits additional ubiquitination (3 sites) lying within the first repeat domain (R1) of the Microtubule binding domain (MTBD).

Hierarchical clustering of all identified Tau PTMs indicates that PTMs are added in a sequential and progressive manner in the cortex of both models (Fig. 3C and 3D), as observed in human disease [20]. PTMs present at all time points (blue clusters) are the earliest occurring sites and might trigger the initiation of Tau pathology. The majority of Tau PTMs accumulate progressively in the cortex during tangle formation at 4 months (orange clusters) and at later stages of disease (magenta clusters). This correlation of PTM amount and Tau pathology indicates that hypermodified, hyperphosphorylated Tau is particularly prone to aggregation.

The most affected brain regions in both models (brainstem and hippocampus) generally display the same PTMs as in the corresponding cortical regions. However, the order of occurrence of these pathological Tau PTMs are not temporally resolved as in the cortex because the majority of modifications are observed at 4 months (Fig. S3A and S3B). To obtain time resolved progression data of these tissues more time points are required between 2 and 4 months. The PTM profiles of less affected subcortical regions exhibit variability in the P301S model or a delayed appearance in the P301L model (Fig. S3C and S3D).

### Phosphorylation of the PRD and the C-terminal domain adjacent to the MTBD drives pathology in the P301S and the P301L mouse models

While some PTMs with less than 5% occupancy are detectable by mass spectrometry these PTMs are likely basal PTMs and not important to driving bulk protein aggregation. Thus, FLEXITau was used to determine the quantitative impact of PTMs mapped [21]. This assay measures the extent of modification of Tau peptides providing sequence coverage from N to C termini.

**Fig. 4:**
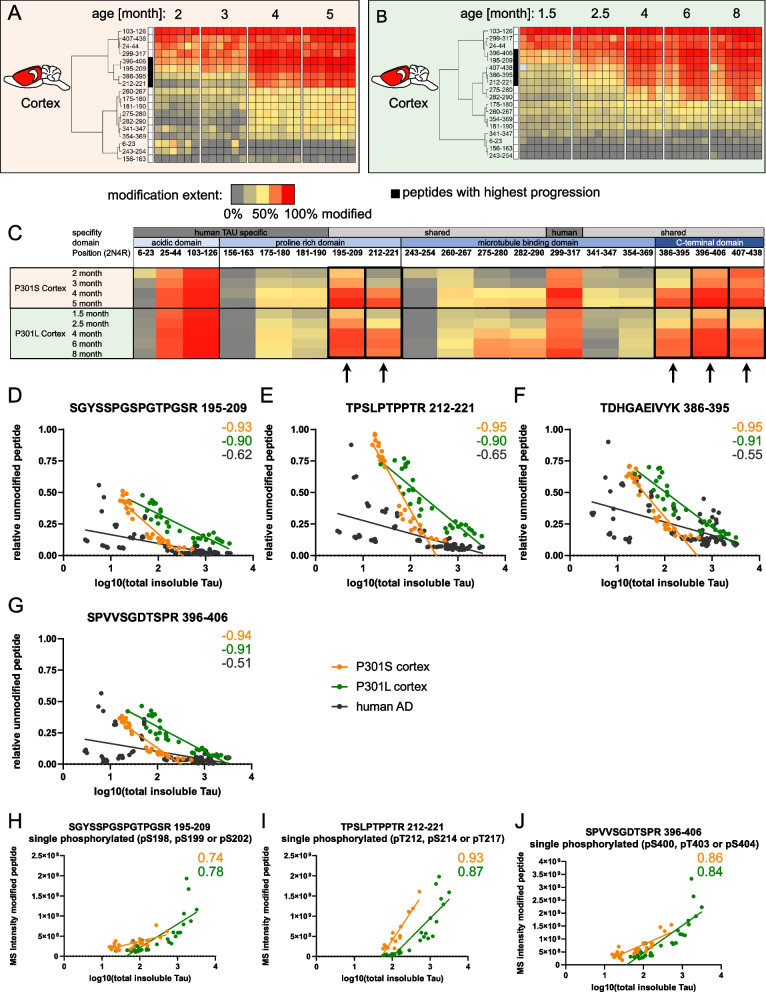
Tau modification extent correlates with Tau pathology kinetics in both models and human AD. **A**-**B** Specific Tau regions and modification sites with high stoichiometry of modification are identified by unbiased data analyses. Euclidean hierarchical clustering of relative amounts of unmodified FLEX-peptide of insoluble Tau derived from the cortex of the P301S (**A**) and P301L model (**B**) shows regions with high modification extent. **C** Average extent of modification of FLEX-peptides per condition from insoluble Tau derived from the cortex of the P301S and P301L model ordered from N- to C-terminus. Arrows indicate regions that become highly modified when aggregates form. **D**-**G** Pearson correlation analyses are performed to study the extent of modification of specific peptides and the amount of insoluble Tau. 195–209 (**D**), 212–221 (**E**), 386–395 (**F**), 396–406 (**G**). This correlation analyses are performed for the cortex of the P301S and P301L model and the BA39 of human AD (human AD data is adapted from [20]). **H**-**J** Pearson correlation of singly phosphorylated peptides with insoluble Tau in the cortex of the P301S and P301L models. Phosphorylated peptides 195–209 (**H**), 212–221 (**I**), 396–406 (**J**) also show a strong association with disease

Hierarchical clustering of the quantitative FLEXITau modification extent data shows that both models exhibit progressive Tau modification during disease which increases drastically with the formation of Tau tangles at 4 months (Fig. 4A and Fig. 4B). The black clusters mark peptides that show the highest fold change in modification extent during progression (3.7 (P301S) and 2.4-fold (P301L)). These peptides progress from no or minimal modification to the highest modification stoichiometries observed. The PTMs likely precipitate the formation of Tau tangles and stabilize these aggregates. The peptides with the highest fold change in modification extent are identical in both models and all brain regions. These high occupancy sites are found in peptides that span the PRD and the C-terminus adjacent to the MTBD (195–209, 212–221, 386–395, and 396–406). The P301L model includes one additional peptide (407–438) (Fig. 4C, Fig. S4E).

Brain regions that show more pathology, such as the brainstem in the P301S model, display earlier and more extensive modification (Fig. S4A and S4B). The less affected subcortical regions show only minimal (P301S) or delayed (P301L) Tau modification stoichiometry (Fig. S4C and S4D). These reinforce the notion that Tau modification contributes to the formation of Tau pathology and that insoluble Tau is formed at different time points during aging.

The contribution of PTMs with the highest fold change of modification to Tau pathology is underscored by a correlation analysis. When peptides with the highest fold change in modification extent are correlated to the amount of insoluble Tau (Fig. 4D-G), Pearson correlation shows that the unmodified Tau peptides decrease at the same rate as Tau pathology increases in both mouse models. At late stages in the mouse disease progression, the modification extent of these peptides is like that observed in late-stage human AD. Of note: the peptides shown in Fig. 4D to 4F are among the top correlating peptides over all analyzed brain regions, mouse models, and in human AD (Fig. S4E and S4F). This similarity suggests that modifications within the mentioned peptides are pivotal for the transition from soluble Tau to Tau tangles and aggregates in both mouse models and human AD.

Within these peptides only phosphorylation sites have been identified in the mouse models (pS199, pS202, pT212, pS214, pT217, pS400, pT403 and pS404). Of these phosphorylation sites some strictly appear with Tau pathology (pT212, pS214, pT217 and pT403). The amount of single phosphorylation sites also correlates with the amount of insoluble Tau in the cortex (Fig. 4H-4J), providing further evidence that these phosphorylation sites are the main drivers of Tau pathology formation in both mouse models and human AD and could therefore serve as targets for therapeutics. In addition, the identified PTMs have been shown to alter Tau structure and solubility in in-vitro experiments [33–36]. Phosphorylation sites that are adjacent to the N-terminal end of the MTBD (212–221, pT212, pS214 or pT217) show the highest correlation and are particularly interesting. Besides the quantified singly phosphorylated peptides, higher phosphorylation states can also contribute to the decrease of unmodified peptides.

### Human carriers of the P301L mutation exhibit minimal Tau pathology

The analyzed transgene mouse models use the P301S and P301L mutation to enhance Tau aggregation. To determine the impact of those mutations on the modification landscape of insoluble Tau in a human context, we analyzed brain samples (frontal gyrus (BA46)) from 5 dementia patients carrying the P301L Tau mutation and 4 healthy aged-matched controls. Human subjects that carry the P301L Tau mutation develop early onset frontotemporal degeneration associated with Tau aggregates, which are found in neurons and glial cells in multiple brain regions of the forebrain [37, 38].

**Fig. 5:**
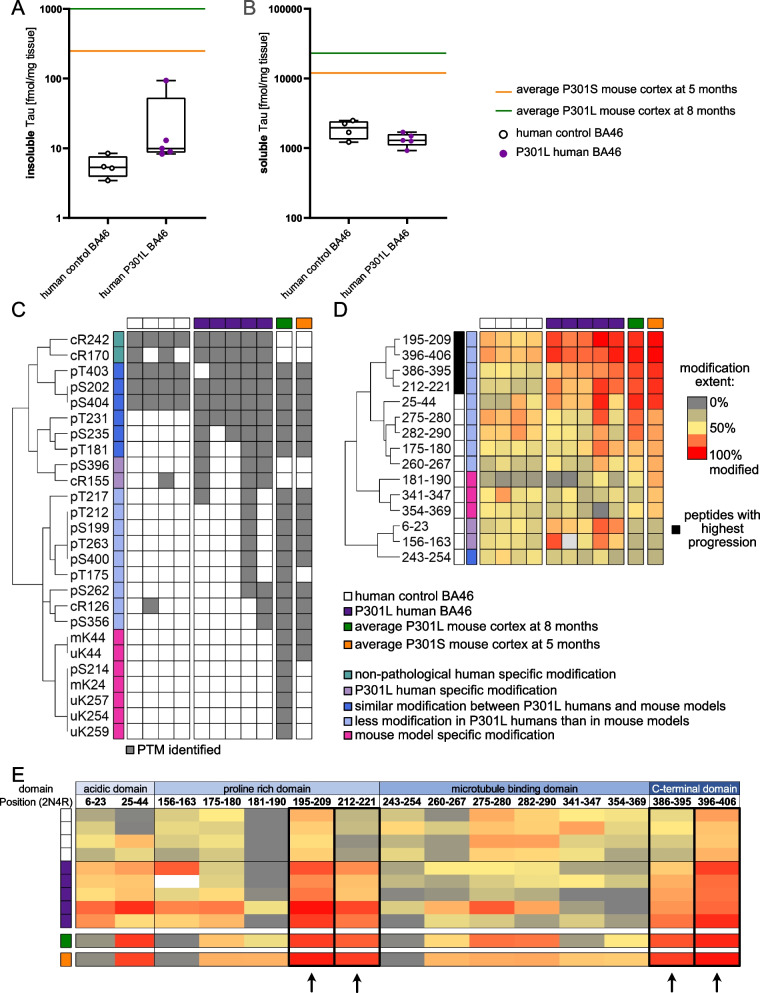
Pathological insoluble Tau of human P301L patients exhibits only minimal modification (Frontal cortex, BA46). **A**, **B** Absolute amount of insoluble (**A**) and soluble (**B**) Tau from P301L patients and healthy controls. **C** Euclidean distance hierarchical clustering of binary PTMs data from insoluble Tau derived from P301L patients and healthy controls in comparison to the cortex in the end stage of the P301S and P301L mouse models. PTMs used for clustering were identified in ≥ 50% of the human biological replicates or were present in one of the mouse models at the last time point. Grey squares indicate the presence of a PTM. **D** Euclidean hierarchical clustering of relative amounts of unmodified FLEX-peptide of insoluble Tau derived from P301L patients and healthy controls in comparison to the cortex in the end stage of the P301S and P301L mouse models. **E** Relative amounts of unmodified FLEX-peptides from insoluble Tau derived from P301L patients and healthy controls ordered from N- to C-terminus controls in comparison to the cortex in the end stage of the P301S and P301L mouse models

The human dementia patients carrying the P301L Tau mutation exhibit 2× more aggregated Tau compared to healthy control subjects (10 fmol/mg tissue, Fig. 5A). The amount of soluble Tau in dementia patients carrying the P301L Tau mutation is decreased by ~ 40% in comparison to the control group (Fig. 5B). This decrease is comparable to late stages of the P301L mouse model and late-stage human AD subjects and could indicate neuronal loss [20]. Like the mouse models insoluble Tau from dementia patients carrying the P301L Tau mutation exhibits mainly phosphorylation (7 sites) however we also observe citrullination sites (3 sites) (using a cutoff of ≥ 50%frequency). In comparison to healthy controls the frequency of 4 phosphorylation and one citrullination sites is increased in dementia patients carrying the P301L Tau mutation. Therefore, these PTMs are considered pathologic (cR155, pT181, pT231, pS235, pS396, Fig. 5C). Most but not all phosphorylation sites of insoluble human P301L Tau are observed in the cortex of mouse models (blue cluster, Fig. 5C). Overall, the insoluble Tau of human dementia patients carrying the P301L Tau mutation remains less modified than the mouse models as shown by the lower amount of accumulated PTMs (light blue and pink clusters, Fig. 5C). These results are consistent with an antibody-based study showing that Tau pathology in human dementia patients carrying the P301L Tau mutation is phosphorylated and not ubiquitinated as observed in AD [37].

The lower modification state of insoluble human P301L Tau is also reflected in the quantitative FLEXITau measurements (Fig. 5D). Most Tau peptides of human dementia patients carrying the P301L Tau mutation are modified in comparison to control, but the modification extent remains lower than the one observed at late stages in both mouse models (light blue cluster). As in the mouse models and human AD, Tau peptides with the highest fold change in modification lie within the PRD and the C-terminus (black cluster/frame, Fig. 5D and E).

In summary human dementia patients carrying the P301L Tau mutation exhibit a similar phosphorylation landscape of insoluble Tau as the mouse models, however, the modification extent is lower which results in less insoluble Tau.

## Discussion

**Fig. 6:**
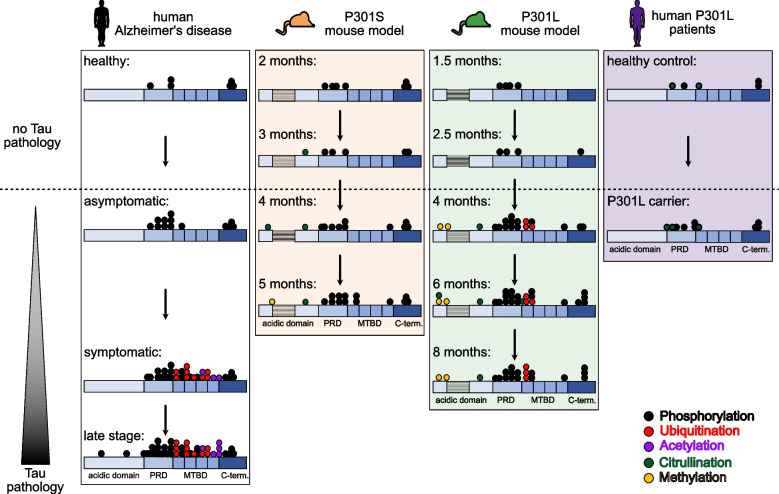
Comparison of the modification landscape of murine and human insoluble Tau. Insoluble Tau of the cortex of P301S and P301L mouse models shows a similar phosphorylation pattern to early stages of human AD and human P301L carriers (human AD data is adapted from [20]). Tau ubiquitination and acetylation of human late-stage AD is not represented in both mouse models. The represented modification sites are documented in table S2. Transverse lines indicate the absence of two N-terminal repeat regions in the human Tau transgene of both mouse models as both models only overexpress 0N4R Tau

Our analysis of the P301S and the P301L model provides a temporally and spatially resolved view of the quantitative and qualitative features of insoluble Tau species in these models. It shows that both mouse models show a strictly ordered progressive accumulation of insoluble Tau as seen in human AD. The qualitative and quantitative analysis of Tau PTMs identifies Tau phosphorylation as the major driver of Tau aggregation in both mouse models. The modification extent of the PRD and the Tau C-terminus correlates tightly with the amount of aggregated Tau. This early sequential phosphorylation of insoluble Tau is reflective of human dementia patients carrying the P301L Tau mutation and early asymptomatic stages of human AD around Braak stage I-III where Tau phosphorylation is the dominant Tau modification (Fig. 6) (Table S2).

Given this observation, the analyzed mouse models are suitable for testing drug candidates that aim to intervene in the early formation of insoluble Tau species promoted by increased phosphorylation of Tau. Insoluble Tau in human AD accumulates additional ubiquitination and acetylation within the MTBD at later symptomatic stages (Braak stages IV-V). However, this important hallmark of human disease is not represented in the mouse models, where either the modification is not observed or found on less important sites. As modifications are a result of the activation of specific pathways, this data suggests that the pathways that lead to these modifications are not activated in the mouse models and the models are therefore unsuitable for mechanistic studies and preclinical drug testing targeting Tau ubiquitination and acetylation.

To be effective animal models of AD, the models need to simulate essential biological processes that contribute to disease in human patients [39]. As non-familial human AD is largely a polygenic, sporadic disease affected by stressors including age, genetics, gender, lifestyle, and environmental factors, the mouse models need to factor in these stressors as initiators and drivers of disease. The current mouse models of Tau pathology use Tau overexpression and risk mutations to induce disease. These models develop pathology that is reflective of familial Tauopathies (Frontotemporal Dementia) that are associated with mutations of Tau (FTLD-17) and differ from sporadic human AD.

More systems-wide analyses are needed to understand the precise functional relationships between global molecular changes and biological phenotypes, in both human AD and mouse models. Also, a detailed analysis of the human disease will help to identify the upstream molecular features that are critical for the formation of human Tau pathology. These essential features of human disease then need to be transferred into the mouse to build reflective models that simulate the full spectrum of human Tau pathology which are needed for successful preclinical drug development.

## Conclusion

AD mouse models that use overexpression of human Tau using risk mutations are reflective models of Tau phosphorylation as seen in familial early onset Tauopathies and early stages of human AD. Therefore, the mouse models are a suitable tool for testing drug candidates that aim to intervene in the early formation of insoluble Tau species promoted by increased phosphorylation of Tau. However, further research is needed to create models that simulate are more complete spectrum of human AD including Tau ubiquitination and acetylation.

### Supplementary Information

**Additional file 1: Figure S1. **Comparison of the FLEXITau assay to AT100 staining. **A** Average area covered by AT100 during disease progression in the cortex of both mouse models (mean +- SD). **B** Pearson correlation analyses of the absolute amount of insoluble Tau derived from the FLEXITau assay and the area covered by AT100 in the cortex of the P301S model. Comparison was performed between the two brain hemispheres of the same animal. **C** Pearson correlation analyses of the absolute amount of insoluble Tau derived from the FLEXITau assay and the area covered by AT100 in the cortex of the P301L model. Comparison was performed between the two brain hemispheres of the same animal.

**Additional file 2.** **Figure S2**. MS intensity of the APP protein in the soluble fraction. **A** Amount of APP in the soluble fraction of the cortex of the P301S model during progression (mean +- SD). **B** Amount of APP in the soluble fraction of the cortex of the P301L model during progression (mean +- SD). As the different models/species were analyzed in different experiments, a quantitative comparison of the results between the experiments is not possible.

**Additional file 3.** **Figure S3**. PTM analyses of pathological Tau during the progression in P301S and P301L mouse models. **A, C** Euclidean hierarchical clustering of binary PTMs data from pathological Tau derived from the P301S brain stem (**A**) and subcortical region (**C**). **B, D** Euclidean hierarchical clustering of binary PTMs data from pathological Tau derived from the P301L hippocampus (**B**) and subcortical region (**D**). PTMs that were used for clustering were found in at least 50% of the biological replicates in at least one time point. Grey squares indicate the presence of a PTM. Annotated clusters represent PTMs identified at all time points (blue), pathological PTMs appearing with tangle formation (orange) and PTMs appearing before (yellow) or after (magenta) the onset of tangle formation.

**Additional file 4**. **Figure S4**. Analysis of the quantitative modification extent (FLEXITau) and Pearson correlation identify regions and modifications of Tau that drive pathology in both models and human AD. **A, C** Euclidean hierarchical clustering of relative amounts of unmodified FLEX-peptide of pathological Tau derived from the P301S brain stem (**A**) and subcortical region (**C**). **B, D** Euclidean hierarchical clustering of relative amounts of unmodified FLEX-peptide of pathological Tau derived from the P301L hippocampus (**B**) and subcortical region (**D**). **E** Average relative amounts of unmodified FLEX-peptides per condition from pathological Tau derived from the P301S brain stem and subcortical region and the P301L hippocampus and subcortical region ordered from N- to C-terminus. **F** Pearson correlation between the amount of unmodified FLEX-peptide and logarithmic amount of pathological Tau ordered from N- to C-terminus for the P301S brain stem and subcortical region and the P301L hippocampus and subcortical region.A legend is provided for the extent of modification and the Pearson correlation coefficient of each peptide. And the in average top 5 correlating peptides.

**Additional file 5**: **Table S1.** Overview of patient demographics of human BA46 tissue samples.

**Additional file 6**: **Table S2.** Overview of PTMs of insoluble Tau species identified at different disease stages of human AD [20], the P301S and the P301L mouse model and human dementia patients carrying the P301L Tau mutation.

## Data Availability

The datasets generated during the current study are available via ProteomeXchange with the dataset identifier PXD033965.

## Abbreviations

AD: Alzheimer’s disease
FTLD: Frontotemporal dementia
LC: Liquid chromatography
MS: Mass spectrometry
MTBD: Microtubule binding domain
PRD: Proline rich domain
PTM: Post translational modification
