# Supplementary figures and images for: Common mouse models of tauopathy reflect early but not late human disease
Source: Mol Neurodegener. 2023 Feb 2;18:10. doi: 10.1186/s13024-023-00601-y (PMC9893608; doi:10.1186/s13024-023-00601-y)

A

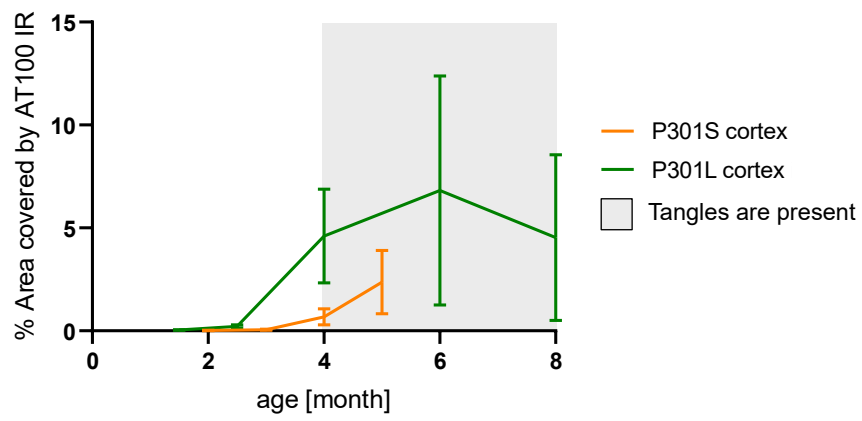

B

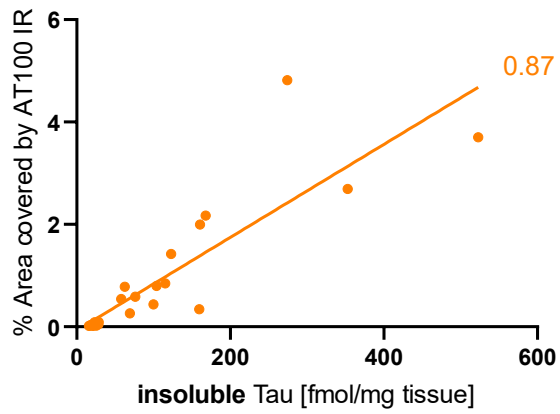

C

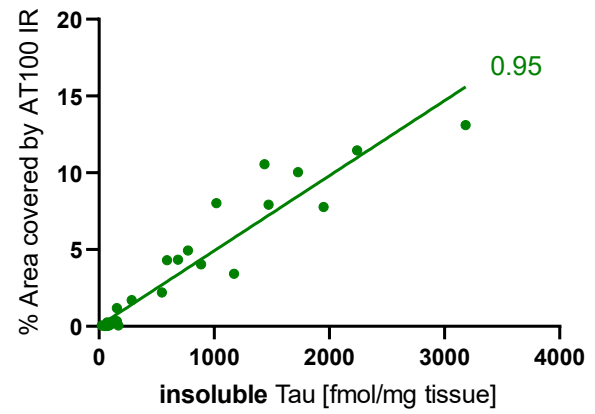

Supplement: Supplementary file 1 — Additional file 1: Figure S1. Comparison of the FLEXITau assay to AT100 staining. A Average area covered by AT100 during disease progression in the cortex of both mouse models (mean +- SD). B Pearson correlation analyses of the absolute amount of insoluble Tau derived from the FLEXITau assay and the area covered by AT100 in the cortex of the P301S model. Comparison was performed between the two brain hemispheres of the same animal. C Pearson correlation analyses of the absolute amount of insoluble Tau derived from the FLEXITau assay and the area covered by AT100 in the cortex of the P301L model. Comparison was performed between the two brain hemispheres of the same animal. [file 13024_2023_601_MOESM1_ESM.pdf]

A

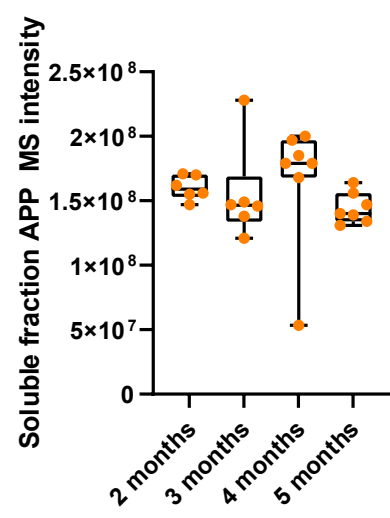

B

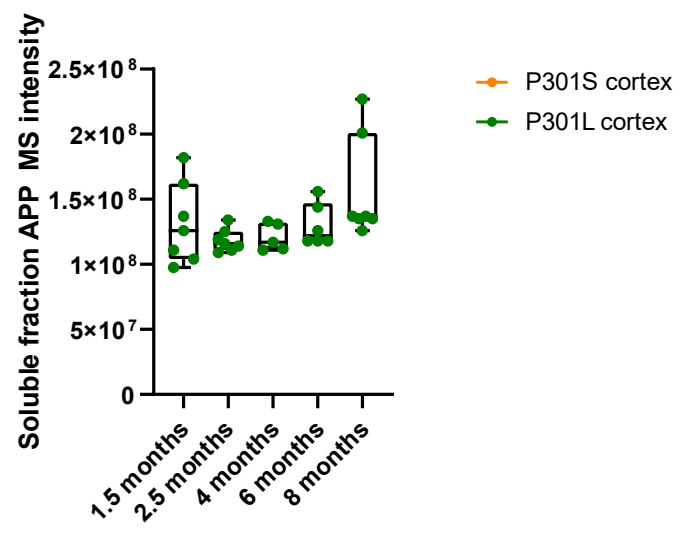

Supplement: Supplementary file 2 — Additional file 2. Figure S2. MS intensity of the APP protein in the soluble fraction. A Amount of APP in the soluble fraction of the cortex of the P301S model during progression (mean +- SD). B Amount of APP in the soluble fraction of the cortex of the P301L model during progression (mean +- SD). As the different models/species were analyzed in different experiments, a quantitative comparison of the results between the experiments is not possible. [file 13024_2023_601_MOESM2_ESM.pdf]

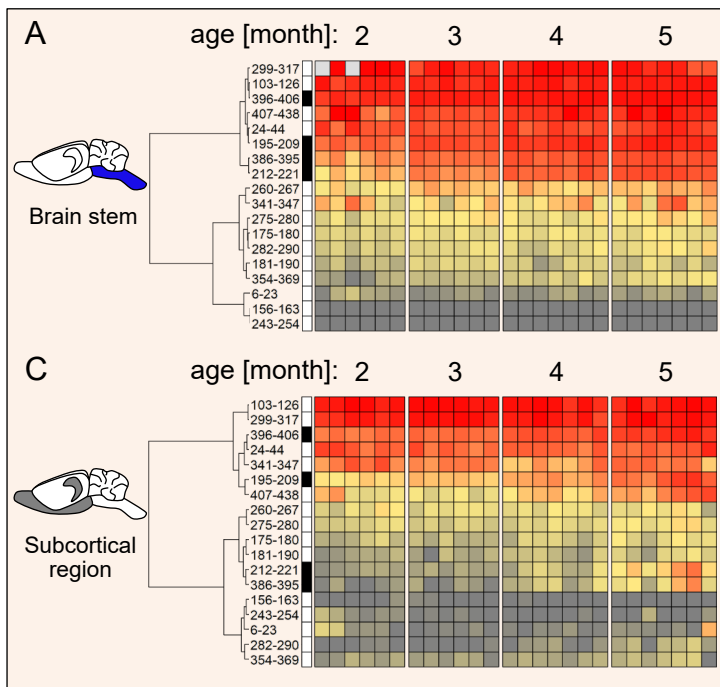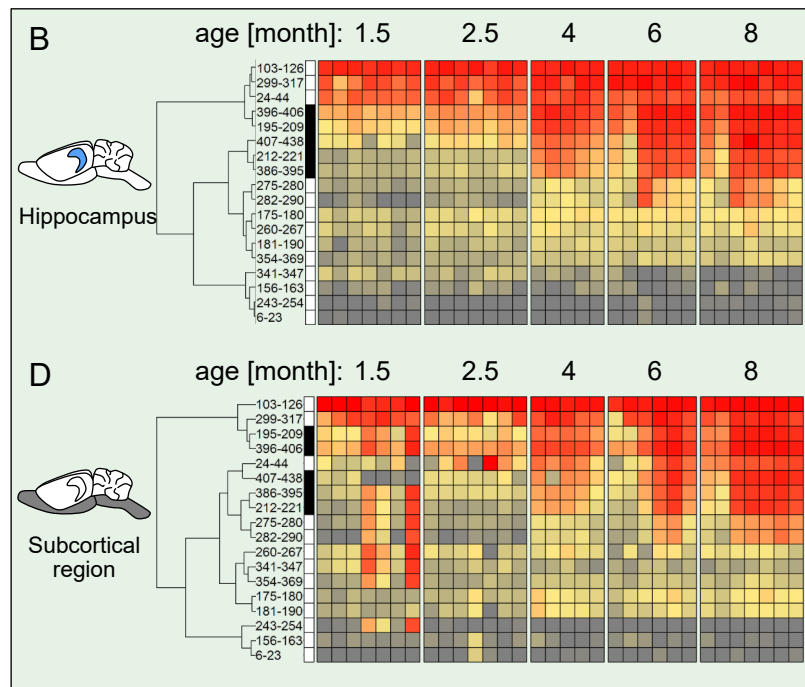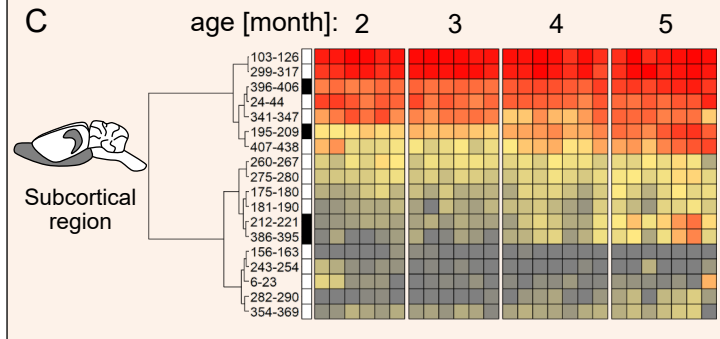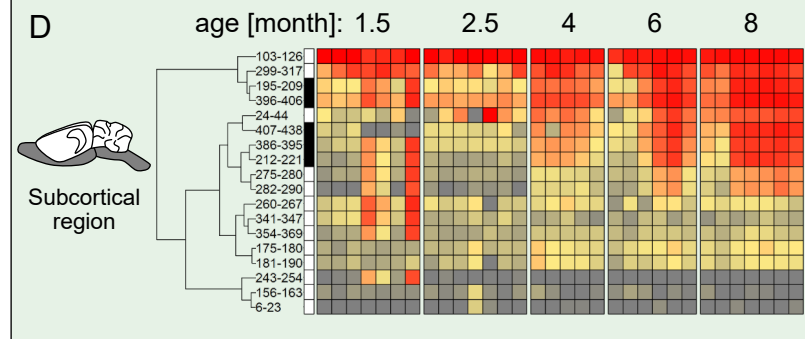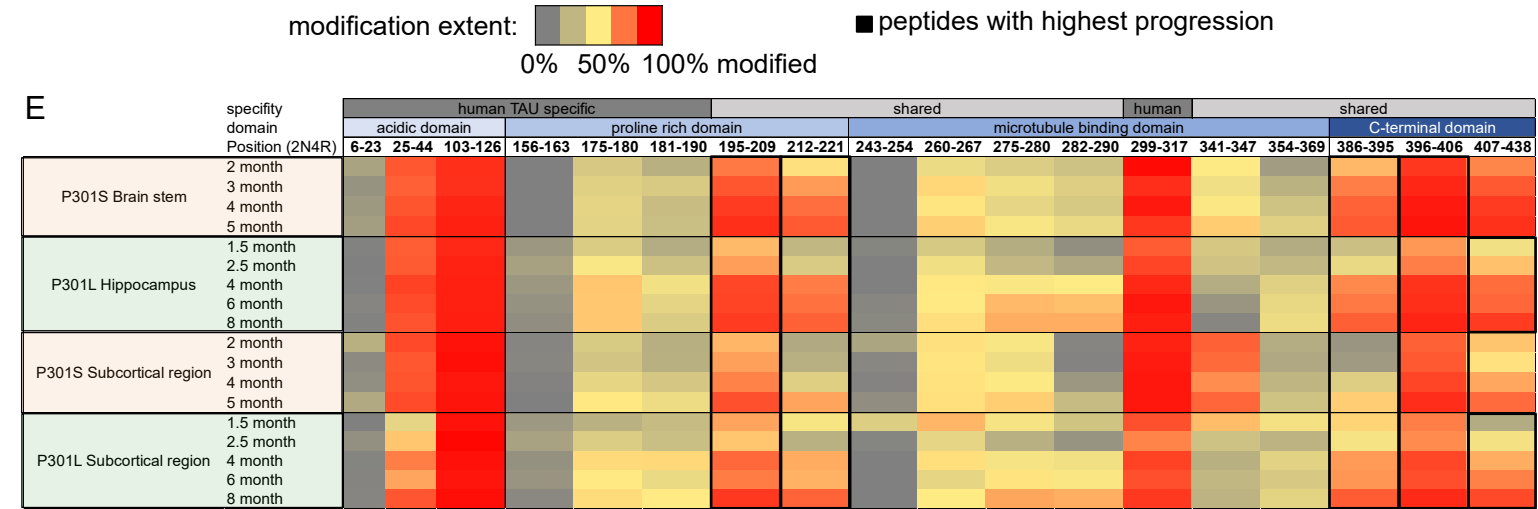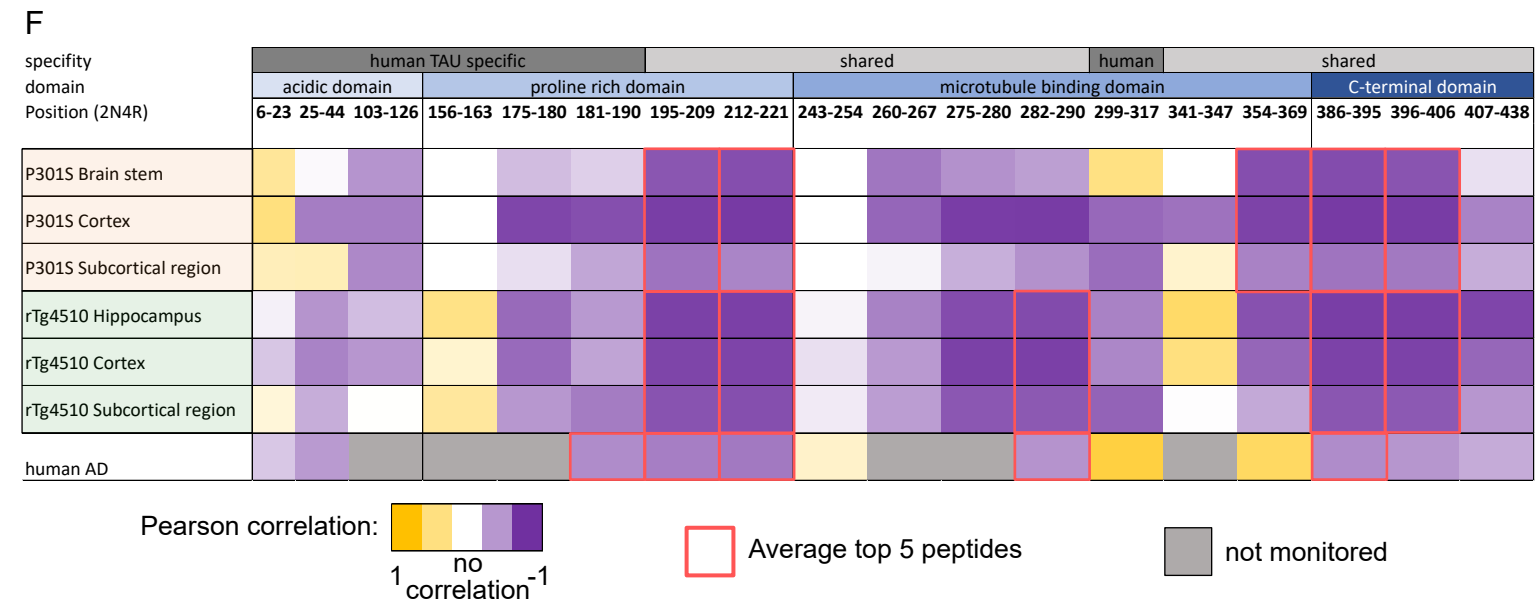

Supplement: Supplementary file 4 — Additional file 4. Figure S4. Analysis of the quantitative modification extent (FLEXITau) and Pearson correlation identify regions and modifications of Tau that drive pathology in both models and human AD. A, C Euclidean hierarchical clustering of relative amounts of unmodified FLEX-peptide of pathological Tau derived from the P301S brain stem (A) and subcortical region (C). B, D Euclidean hierarchical clustering of relative amounts of unmodified FLEX-peptide of pathological Tau derived from the P301L hippocampus (B) and subcortical region (D). E Average relative amounts of unmodified FLEX-peptides per condition from pathological Tau derived from the P301S brain stem and subcortical region and the P301L hippocampus and subcortical region ordered from N- to C-terminus. F Pearson correlation between the amount of unmodified FLEX-peptide and logarithmic amount of pathological Tau ordered from N- to C-terminus for the P301S brain stem and subcortical region and the P301L hippocampus and subcortical region.A legend is provided for the extent of modification and the Pearson correlation coefficient of each peptide. And the in average top 5 correlating peptides. [file 13024_2023_601_MOESM4_ESM.pdf]
