# Supplementary material for: Common mouse models of tauopathy reflect early but not late human disease
Source: Mol Neurodegener. 2023 Feb 2;18:10. doi: 10.1186/s13024-023-00601-y (PMC9893608; doi:10.1186/s13024-023-00601-y)
Supplement: Supplementary file 3 — Additional file 3. Figure S3. PTM analyses of pathological Tau during the progression in P301S and P301L mouse models. A, C Euclidean hierarchical clustering of binary PTMs data from pathological Tau derived from the P301S brain stem (A) and subcortical region (C). B, D Euclidean hierarchical clustering of binary PTMs data from pathological Tau derived from the P301L hippocampus (B) and subcortical region (D). PTMs that were used for clustering were found in at least 50% of the biological replicates in at least one time point. Grey squares indicate the presence of a PTM. Annotated clusters represent PTMs identified at all time points (blue), pathological PTMs appearing with tangle formation (orange) and PTMs appearing before (yellow) or after (magenta) the onset of tangle formation. [file 13024_2023_601_MOESM3_ESM.pdf]

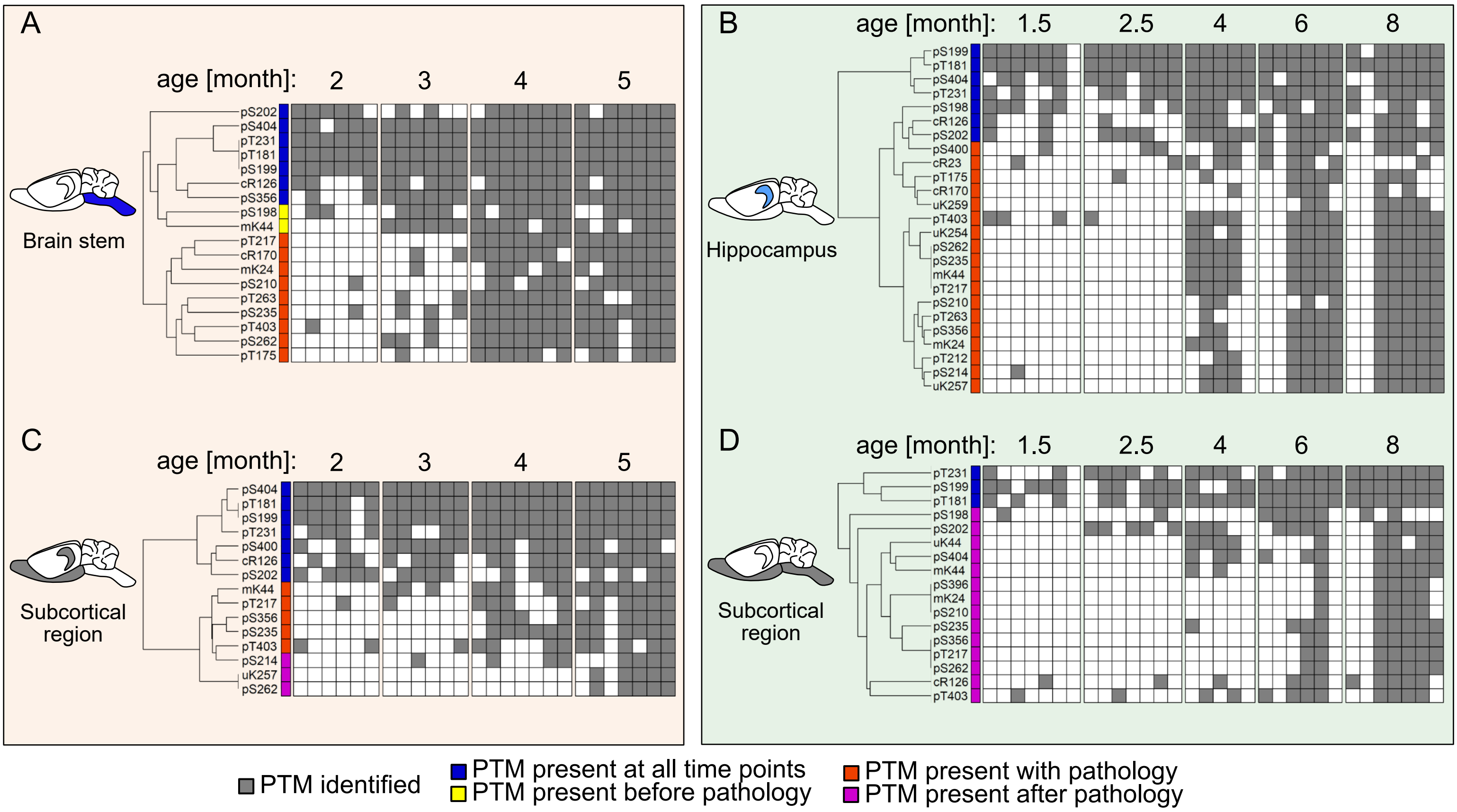
A
age [month]: 2 3 4 5
pS202
pS404
pT231
pT181
pS199
cR126
pS356
pS198
mK44
pT217
cR170
mK24
pS210
pT263
pS235
pT403
pS262
pT175
Brain stem
B
age [month]: 1.5 2.5 4 6 8
pS199
pT181
pS404
pT231
pS198
cR126
pS202
pS400
cR23
pT175
cR170
uK259
pT403
uK254
pS262
pS235
mK44
pT217
pS210
pT263
pS356
mK24
pT212
pS214
uK257
Hippocampus
C
age [month]: 2 3 4 5
pS404
pT181
pS199
pT231
pS400
cR126
pS202
mK44
pT217
pS356
pS235
pT403
pS214
uK257
pS262
Subcortical region
D
age [month]: 1.5 2.5 4 6 8
pT231
pS199
pT181
pS198
pS202
uK44
pS404
mK44
pS396
mK24
pS210
pS235
pS356
pT217
pS262
cR126
pT403
Subcortical region
PTM identified
PTM present at all time points
PTM present before pathology
PTM present with pathology
PTM present after pathology
