# Supplementary material for: Common mouse models of tauopathy reflect early but not late human disease
Source: Mol Neurodegener. 2023 Feb 2;18:10. doi: 10.1186/s13024-023-00601-y (PMC9893608; doi:10.1186/s13024-023-00601-y)
Supplement: Supplementary file 5 — Additional file 5: Table S1. Overview of patient demographics of human BA46 tissue samples. [file 13024_2023_601_MOESM5_ESM.docx]

**Table S1** Overview of patient demographics of human BA46 tissue samples.

| Genotype | Sex | Clinical diagnosis | Braak stage | Age at death | Mean age of death (SD) |
| --- | --- | --- | --- | --- | --- |
| control | Female | cerebrovascular disease | 0 | 81 | 83.0 (9.0) |
| control | Male | - | I | 71 |  |
| control | Male | - | I | >=90 |  |
| control | Female | - | I | >=90 |  |
| P301L | Male | FTLD | 0 | 61 | 58.2 (15.4) |
| P301L | Female | FTD | IV | 71 |  |
| P301L | Male | FTD TAU | I | 56 |  |
| P301L | Male | PICKS |  | 33 |  |
| P301L | Male | AD |  | 70 |  |
