# Supplementary material for: Common mouse models of tauopathy reflect early but not late human disease
Source: Mol Neurodegener. 2023 Feb 2;18:10. doi: 10.1186/s13024-023-00601-y (PMC9893608; doi:10.1186/s13024-023-00601-y)
Supplement: Supplementary file 6 — Additional file 6: Table S2. Overview of PTMs of insoluble Tau species identified at different disease stages of human AD [20], the P301S and the P301L mouse model and human dementia patients carrying the P301L Tau mutation. [file 13024_2023_601_MOESM6_ESM.docx]

**Table S2** Overview of PTMs of insoluble Tau species identified at different disease stages of human AD [20], the P301S and the P301L mouse model and human dementia patients carrying the P301L Tau mutation.

| **Human AD** | **P301S mouse model** | **P301L mouse model** | **Human P301L** |
| --- | --- | --- | --- |
| **control:**  pT181, pT231, pS235, pS400, pT403, pS404 | **2 months:**  pT181, pS199, pS202, pT231, pS400, pT403, pS404 | **1.5 months:**  pT181, pS199, pS202, pT231 | **Control:**  cR170, pS202, cR242, pT403, pS404 |
| **asymptomatic:**  pS199, pT181, pS199, pS202, pT212, pT217, pT231, pS235, pS237, pS262, pS396, pS400, pT403, pS404 | **3 months:**  cR126, pT181, pS199, pT231, pS400, pS404 | **2.5 months:**  pT181, pS199, pT231, pS400 | **P301L carrier:**  cR155, cR170, pT181, pS202, pT231, pS235, cR242, pS396, pT403, pS404 |
| **Symptomatic:**  pT175, pT181, pS199, pS202, pS210, pT212, pS214, pT217, pT231, pS235, pS237, uK254, uK259, pS262,pT263, uK267, uK274, uK281, pS289, uK290, pS305, uK311, aK311, uK317, uK321, aK252, aK369, pS356, pS396, pS400, pT403, pS404 | **4 months:**  cR23, cR126, pT181, pS199, pS202, pT217, pT231, pS235, pS356, pS400, pT403, pS404 | **4 months:**  mK24, mK44, cR126, pT175, pT181, pS199, pS202, pT212, pS214, pT217, pT231, pT235, uK254, uK257, pS262, uK267, pS356, pT403, pS404 |  |
| **symptomatic late stages:** pS55, pS113, pT153, pT175, pT181, pS191, pS198, pS199, pS202, pT205, pS210, pT212, pS214, pT217, pT231, pS235, pS237, uK254, uK257, uK259, pS262, pT263, uK267, uK274, uK281, pS289, uK290, pS293, pS305, uK311, aK311, uK317, uK321, aK252, pS356, aK369, aK270, aK375, pS396, pS400, pT403, pS404 | **5 months:**  mK44, uK44, cR126, pT181, pS199, pS202, pT212, pT217, pT231, pS235, pS262, pT263, pS356, pS400, pT403, pS404 | **6 months:**  cR23, mK24, mK44, uK44, cR126, pT175, pT181, pS199, pS202, pS210, pT212, pS214, pT217, pT231, pT235, cR242, uK254, uK257, pS262, pT263, uK267, pS356, pS396, pS400, pT403, pS404 |  |
|  |  | **8 months:**  mK24, mK44, uK44, cR126, pT175, pT181, pS199, pS202, pT212, pS214, pT217, pT231, pT235, uK254, uK257, uK259, mK259, pS262, pT263, pS356, pS400, pT403, pS404 |  |
|  |  |  |  |
